# In Vitro Inhibition of Endoplasmic Reticulum Stress: A Promising Therapeutic Strategy for Patients with Crohn’s Disease

**DOI:** 10.3390/cells14040270

**Published:** 2025-02-13

**Authors:** Bruno Lima Rodrigues, Lívia Bitencourt Pascoal, Lívia Moreira Genaro, Leonardo Saint Clair Assad Warrak, Beatriz Alves Guerra Rodrigues, Andressa Coope, Michel Gardere Camargo, Priscilla de Sene Portel Oliveira, Maria de Lourdes Setsuko Ayrizono, Lício Augusto Velloso, Raquel Franco Leal

**Affiliations:** 1 Inflammatory Bowel Disease Research Laboratory, Colorectal Surgery Unit, Gastrocenter, School of Medical Sciences, University of Campinas (Unicamp), São Paulo 13083-878, Brazil; 2 Laboratory of Cell Signaling, Obesity and Comorbidities Research Center, School of Medical Sciences, University of Campinas (Unicamp), São Paulo 13083-864, Brazil

**Keywords:** Crohn’s disease, endoplasmic reticulum stress, unfolded protein response

## Abstract

Background: Crohn’s disease (CD) is an inflammatory bowel disease marked by an abnormal immune response and excessive pro-inflammatory cytokines, leading to impaired protein processing and endoplasmic reticulum (ER) stress. This stress, caused by the accumulation of misfolded proteins, triggers the unfolded protein response (UPR) through IRE1/Xbp-1, PERK/eIF2α, and ATF6 pathways, which are linked to intestinal inflammation. This study aimed to investigate ER stress in CD patients’ intestinal mucosa and evaluate phenylbutyrate (PBA) as an ER stress inhibitor. Methods: Colon biopsies from CD patients and controls were cultured under five conditions, including 4-PBA treatments. Real-time PCR, cytokine level, and immunohistochemistry were performed. Results: Immunohistochemistry revealed that ER stress was activated in CD patients’ intestinal epithelial cells and lamina propria cells. PERK/eIF2α, but not IRE1/Xbp-1 or ATF6, was upregulated in CD patients compared to controls. UPR-related genes (STC2, CALR, HSPA5, HSP90B1) were also elevated in CD patients. PBA treatment significantly reduced ER stress and UPR markers while decreasing apoptotic markers like DDIT3. Pro-inflammatory cytokines, such as IL-1β, IL-6, IL-17, TNF- α, and sCD40L, were significantly reduced after PBA treatment. Conclusion: ER stress and UPR pathways are activated in CD colonic mucosa, and PBA reduces these markers, suggesting potential therapeutic benefits for CD-related inflammation.

## 1. Introduction

Crohn’s disease (CD) is a multifactorial condition in which a maladaptive immune response to gut-commensal bacteria leads to cytokine imbalance in genetically predisposed individuals [1]. Most of the patients present an inflammatory phenotype, but given its segmental and transmural characteristics, it is expected that serious complications, such as strictures and fistulas, might develop over time, resulting in surgical interventions [2]. Although CD is more common in developed regions, such as the United States of America and most countries in the north of Europe, developing countries have an increasing incidence [3,4].

Once the disease is activated, various molecular pathways are triggered, maintaining the disease and influencing the treatment response. One of these pathways is associated with the accumulation of unfolded protein in the lumen of the endoplasmic reticulum (ER), inducing a complex response named unfolded protein response (UPR) [5]. A combination of factors that disrupt ER homeostasis can lead to unfolded protein accumulation in this organelle, such as increased protein synthesis (pro-inflammatory cytokines, for example); oxidative stress, a hallmark of CD, that can damage proteins and interfere with their proper folding; malabsorption of nutrients, common in CD, can lead to deficiencies in essential cofactors required for protein folding; and genetic mutations associated with CD can affect genes involved in protein folding and ER response, further contributing to the accumulation of unfolded proteins [6,7]. Subsequent events of UPR, called ER stress, aim to preserve cell integrity, reestablishing ER homeostasis through the activation of three main pathways: transmembrane inositol-requiring enzyme-1α and β (IRE1α and β), protein kinase-like ER kinase (PERK), and activating transcription factor 6 (ATF6) [8].

**Figure 1:**
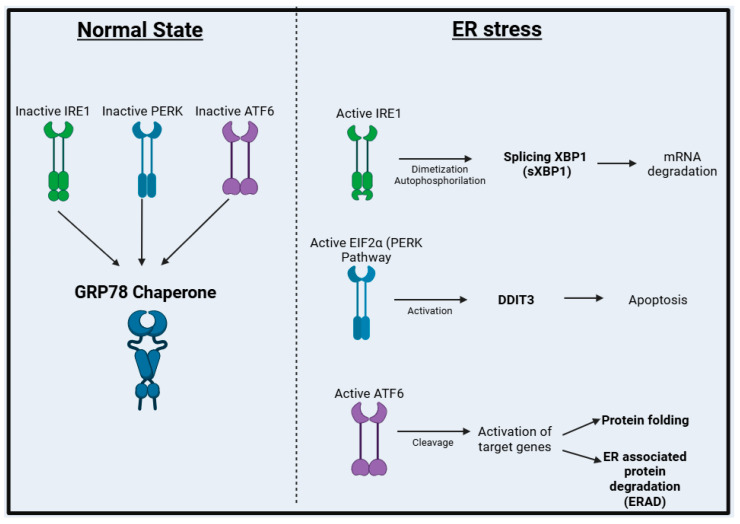
**ER stress signaling pathways: IRE1, PERK, and ATF6.** IRE1, PERK, and ATF6 remain inactive and bound to the chaperone GRP78 under normal conditions. Under endoplasmic reticulum (ER) stress, these proteins dissociate and trigger the UPR response: IRE1 undergoes dimerization and autophosphorylation, activating the transcription factor XBP1; PERK phosphorylates eIF2α, promoting protein folding and apoptosis via DDIT3; and ATF6 is cleaved, activating genes involved in protein folding and ER-associated degradation (ERAD).

During the normal state, IRE1, PERK, and ATF6 remain inactivated and bound to the chaperone GRP78. Once ER stress is initiated, these proteins are released and start a cascade of reactions that results in UPR activation [9,10]; IRE1 undergoes dimerization and autophosphorylation and promotes splicing of X-box-binding protein 1 (XBP1), a transcription factor responsible for promoting mRNA degradation [11]. The phosphorylation of eIF2α (accountable for activating the PERK pathway) results in protein folding. It induces apoptosis via activation of DNA damage-inducible transcript 3 (DDIT3) [12,13], whereas ATF6 undergoes cleavage, which causes the activation of target genes responsible for protein folding or ER-associated protein degradation (ERAD) (Figure 1) [14,15].

Although ER stress is an important defense mechanism in response to a toxic state in the lumen of ER, prolonged activation is considered harmful to the cell [10,16]. Given that UPR induces the activation of IκB kinase (IKK)/NF-κB and c-Jun N-terminal Kinase (JNK)/AP1, which leads to transcriptional activation of pro-inflammatory cytokines genes such as TNF-α, IL-1β, and IL-6, chronic ER stress leads to the exacerbation and perpetuation of inflammation in the gut that contributes to the progression of CD [5].

ER stress has been linked to inflammatory bowel disease (IBD), mainly in epithelial intestinal cells, which can be associated with the activation of the immune response and intestinal dysbiosis, critical factors in the maintenance of CD [9,17]. The intestinal epithelium of mice deficient in the production of Xbp1, ATF6a, and P58 have a high expression of CHOP (C/EBP homologous protein), one of the products of PERK activation [18]. IRE1α knockout mice (IRE1α-/-) are more susceptible to colitis induction with the use of dextran sulfate sodium (DSS) and to the apoptotic process caused by ER stress activation [19]. In addition, in the last few years, our group published studies showing that EIF2AK3 and ERN1, genes that encode PERK and IRE1 activation, respectively, were upregulated in intestinal mucosa from patients with CD but not in the mesenteric adipose tissue [20], and also in patients with ulcerative colitis, another form of IBD [21].

Targeting ER stress pathways may offer new therapeutic strategies for CD. The search for a compound able to block ER stress and UPR in chronic conditions has become a goal in diverse fields of medicine. 4-Phenylbutyrate (4-PBA) is a low molecular weight chemical chaperone tested in animal models of obesity to modulate UPR [22] and used to treat urea cycle disorders [23]. In vitro studies reported that 4-PBA can suppress ER stress [24,25], and its use for humans has been reported to be safe [26]. Although PBA has effectively reduced DSS-induced colitis in mice [27,28], its proprieties as ER stress suppressors have not yet been studied in IBD. Therefore, this study aims to evaluate the effectiveness of PBA in reducing, the activation of ER stress pathways in intestinal mucosa biopsies from patients with active CD in vitro.

## 2. Materials and Methods

### 2.1. Patients and Ethics Statement

Samples from the affected colon were collected from CD patients (CD group) who had undergone colonoscopy exams. Disease activity was assessed using the clinical Crohn’s Disease Activity Index (CDAI) and Crohn’s Disease Endoscopic Index of Severity (CDEIS). Patients with CDEIS ≥ 5 or the presence of larger ulcers in at least one intestine segment were included; those who did not present endoscopic active disease in the colon were excluded. The control group comprised patients who underwent colonoscopy for reasons other than IBD. All participants read and signed a written informed consent form approved by the local Ethics Committee.

Table 1 shows patients’ clinical and demographic data included in the immunohistochemistry analysis. Table 2 shows patients’ clinical and demographic data included in the transcriptional analysis and multiplex assay. Patients were classified according to the Montreal classification. The age of diagnosis (A) was classified as A1: Diagnosis at 16 years or younger; A2: Diagnosis between 17 and 40; or A3: Diagnosis at 40 or older. The location of disease (L) was classified as L1: ileal involvement (small intestine); L2: colonic involvement; L3: ileocolonic involvement (both small and large intestines); or L4: upper gastrointestinal involvement. Behavior of disease (B) was classified as B1: non-stricturing, non-penetrating disease; B2: stricturing disease; or B3: penetrating disease [29]. Most patients included in the study had the A2 classification, had a colonic or ileocolonic disease, and had an inflammatory or stricturing form of the disease. These data follow the most frequent characteristics of CD observed in the literature that address the epidemiology of this affection [30,31,32,33].

### 2.2. Immunochemistry

Intestinal mucosa samples from CD patients and controls were embedded in paraffin and sectioned at 4 µm. The sections were deparaffinized with xylene (tree washes 20 min each), 100% ethanol (three washes 3 min each), 80% ethanol (3 min), 50% ethanol (3 min), and deionized water (three minutes). The antigen retrieval was performed using a citrate buffer (pH 6.0) at 95 °C for 20 min. A 3% hydrogen peroxide solution (H_2_O_2_ 10 vol) was applied to block endogenous peroxidase activity, followed by washes in phosphate-buffered saline (PBS, 10 mM, pH 7.4). Blocking with 1% bovine serum albumin (1% BSA), diluted in PBS, was performed as a blocker to reduce nonspecific interactions between antibodies and sample surfaces. Primary antibodies were prepared in a 1% BSA solution (diluted in PBS) and incubated overnight at 4 °C. The antibodies used included anti-phosphor-[Ser51] eIF2a (Abcam, ab32157, rabbit monoclonal), purified anti-Xbp-1 (BioLegend, 658802, mouse), GRP78 (Bioss Antibodies, bs-1219R, rabbit polyclonal), GRP94 (Bioss Antibodies, bs-0194R, rabbit polyclonal), anti-ATF6 (Atlas Antibodies, HPA005935, rabbit), and anti-DDIT3 (BioVision, A1674-100). Signal detection was carried out using an immunoperoxidase detection kit (Vector Laboratories), followed by incubation with a DAB solution (Dako). Slides were rinsed with distilled water, counterstained with hematoxylin, dehydrated through a graded series of alcohol, and mounted using Dako Mounting Medium. Images were captured using a Leica DM4500 microscope equipped with a Leica DFC290 digital camera with control software (Leica Microsystems—Wetzlar).

### 2.3. Explant Culture

The biopsies obtained from intestinal mucosa were cultured in RPMI-1640 medium (Sigma-Aldrich^®^, Darmstadt, Germany), devoid of L-glutamine, and supplemented with 10% fetal calf serum and an antibiotic-antimycotic mixture (Gibco Invitrogen, Thermo Fisher Scientific, Waltham, Massachusetts, USA). The samples were subdivided and subjected to five distinct treatment conditions: control (medium only), vehicle (medium + MilliQ^®^ water, Millipore, Darmstadt, Germany), infliximab at 100 µg/mL (Remicade^®^, Janssen-Cilag, Beerse, Belgium), and 4-PBA (Sigma-Aldrich^®^, Darmstadt, Germany) at concentrations of 5 mM and 10 mM. The 5 mM dose was based on previous studies [34,35,36,37], which have demonstrated that PBA, at this concentration, effectively inhibits markers of ER stress. We also aimed to investigate whether a higher concentration of PBA (10 mM) might yield enhanced inhibitory effects. The cultures were maintained for 6 h in a humidified 5% CO_2_ atmosphere at 37 °C. Following the treatment period, the samples were processed for RNA extraction, and the culture supernatant was collected for quantification of inflammatory cytokines.

### 2.4. RNA Extraction and cDNA Synthesis

Total RNA was extracted from the sample following culture and PBA treatment using the RNeasy Mini Kit (Qiagen, Hilden, Germany, Cat No./ID: 74104) in accordance with the manufacturer’s protocol. RNA purity and concentration were assessed via UV spectrophotometry at 260 nm. cDNA synthesis was performed using the High Capacity cDNA Reverse Transcription Kit (Applied Biosystems, Foster City, CA, USA), following the manufacturer’s instructions. The resulting cDNA was subsequently diluted to the appropriate concentration for efficient amplification of each gene.

### 2.5. Quantitative Real-Time PCR (qPCR)

Real-time quantitative PCR was conducted using extracted RNA and the TaqMan™ system (Applied Biosystems). The following TaqMan primers were utilized: EIF2AK3 (Hs_00178128_m1), ERN1 (Hs_00980095_m1), IL-6 (Hs00174131_m1), TNFa (Hs00174128_m1), and glyceraldehyde-3-phosphate dehydrogenase (GAPDH) (4326317E), all sourced from Applied Biosystems, with a final concentration of 500 nM, per the manufacturer’s recommendations (Applied Biosystems, TaqMan Assays). Gene expression was normalized to GAPDH, a commonly used housekeeping gene known for its stability. In accordance with Life Technologies’ protocol, the FAM™ dye was used as the reporter for target assays, while VIC™ dye was employed for the normalizer assay. Reverse transcription PCR (RT-PCR) was carried out following the manufacturer’s instructions. The RT-PCR amplification comprised an initial denaturation at 95 °C for 3 min, followed by 45 cycles of denaturation at 95 °C for 5 s, annealing at 60 °C for 5 s, and extension at 60 °C for 30 s, concluding with a final incubation at 60 °C for 1 min. Each PCR reaction contained 40 ng of cDNA, 2.5 μL of each specific primer, TaqMan Universal Master Mix (4369016, Life Technologies, Carlsbad, Califórnia, EUA), and RNase-free water, with a final volume of 10 μL. Gene expression analysis was performed using the Applied Biosystems StepOne™ detection system, and relative gene expression was calculated using the delta-delta Ct method. The rt-PCR was performed in biological duplicates. Given the difficulty of selecting the ideal patients and harvesting and processing the samples, the experiments were performed only once.

### 2.6. Cytokines Quantification

The concentrations of IL-1β, IL-4, IL-6, IL-10, IL-17A, IL-17F, IL-21, IL-22, IL-23, IL-25, IL-31, IL-33, CD40L, IFN-γ, and TNF-α were quantified from the supernatant of explant cultures using the Bio-Plex Pro Human Th17 Cytokine Assay Kit (catalog number 171AA001M, Bio-Rad Laboratories, Hercules, CA, USA), a multiplex assay, following the manufacturer’s protocol. In brief, assay plates were pre-wetted with assay buffer and washed twice with wash buffer. Coupled magnetic beads were then added to the 96-well plate, along with serial dilutions of the reconstituted standard and the experimental samples. The mixture was incubated in the dark at room temperature on an orbital shaker for 60 min. After incubation, detection antibodies diluted in antibody diluent were added to the wells and incubated for an additional 30 min at room temperature, followed by washing and the addition of streptavidin-PE for 10 min at room temperature. After a final wash, the samples were analyzed on the Luminex instrument using Bio-Plex Manager software (version 6.0) at LacTAD, Unicamp.

### 2.7. Statistics

Statistical analyses were carried out using nonparametric tests. The Shapiro test was conducted to assess statistical normality. Once normality was established, a *t*-test and Mann–Whitney test between the groups were performed. A *p*-value less than 0.05 was considered significant.

## 3. Results

### 3.1. Activation of ER Stress in the Intestinal Mucosa of Crohn’s Disease Patients

**Figure 2:**
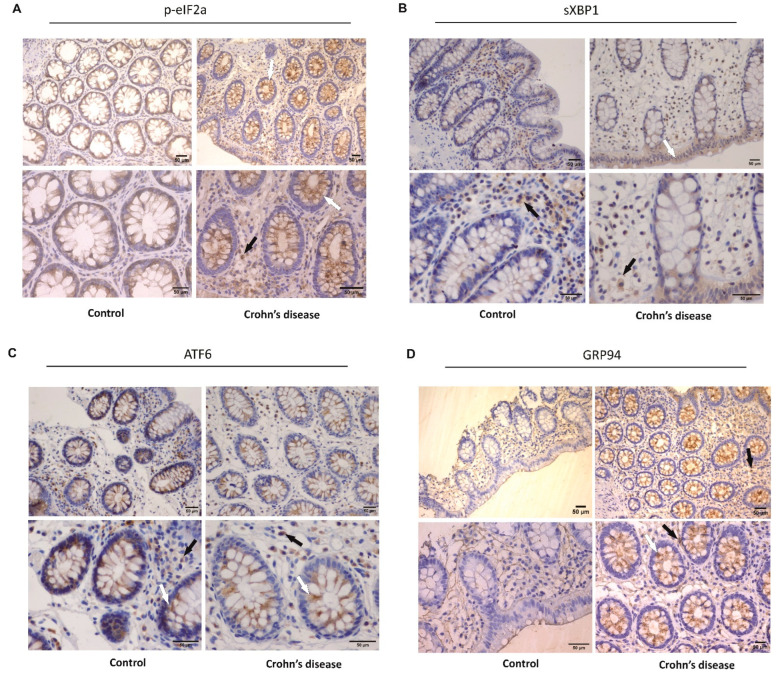
**Activation of ER stress pathways and chaperone modulation in the colonic mucosa of Crohn’s disease patients.** Immunohistochemical analysis of p-eIF2α (**A**), sXBP1 (**B**), ATF6 (**C**), and GRP94 (**D**) was performed on paraffin-embedded slides from the intestinal mucosa of both Crohn’s disease and control groups. The white arrows indicate positive epithelial cells, and the black arrows signalize positive cells from the lamina propria. Scale bar: 50 μm.

To show ER stress activation before the in vitro experiments, we performed immunohistochemistry (IHC) in the intestinal mucosa of eight CD patients and seven non-IBD patients as the control group (Table 1). IHC images revealed positive immunostaining for p-eIF2α and sXBP1 expression in epithelial intestinal cells and cells from the lamina propria of CD patients (Figure 2A,B). In contrast, almost no positive cells were detected in the controls, only sXBP1 expression in the lamina propria cells. Moreover, no differences in the positive immunostaining for ATF6 in CD and control groups were found; positive cells were marked in lamina propria and epithelial cells in both groups (Figure 2C).

An increase in chaperone modulation is often seen after UPR activation. Therefore, we also performed IHC for GRP94. As a result, we observed positive immunostaining for GRP94 in epithelial intestinal cells and cells from lamina propria from CD patients, whereas no positive cells were detected in the control group (Figure 2D).

### 3.2. Blockade of Intestinal Mucosa ER Stress of Crohn’s Disease by a Chemical Chaperone

**Figure 3:**
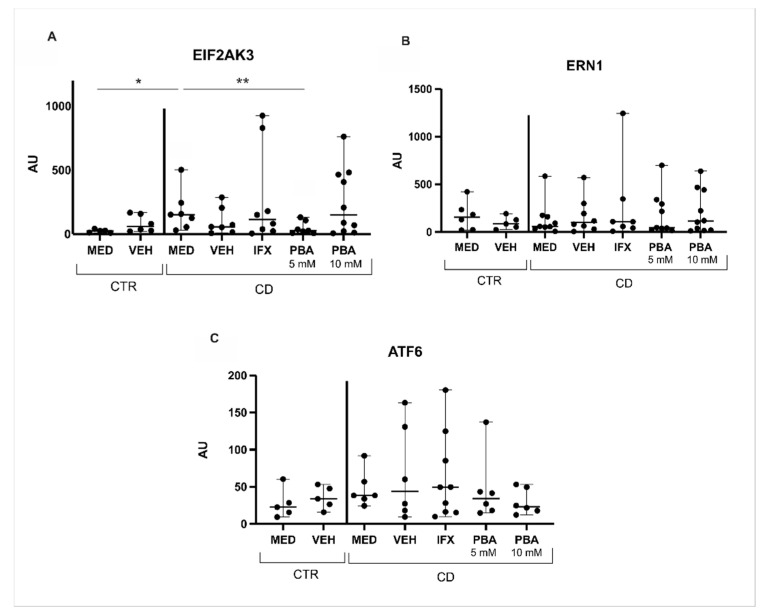
**ER stress activation in the intestinal mucosa of Crohn’s disease patients and PBA inhibition effect in vitro.** (**A**) The EIF2AK3 (PERK pathway) transcriptional level was significantly higher in the MED CD condition than in the MED CTR condition. After treatment with PBA, the EIF2AK3 transcriptional level significantly decreased comparing MED CD to the CD PBA 5 mM condition. (**B**) ERN1 (IRE1 pathway) and (**C**) ATF6 transcriptional levels were similar when comparing the MED CD and MED CTR conditions. Moreover, no differences were observed in the ERN1 transcriptional level after PBA treatment, and neither in ATF6. MED = medium, VEH = vehicle, IFX = infliximab, PBA = 4-phenylbutyrate acid, CD = Crohn’s disease, CTR = control. * *p* = 0.005 vs. MED CTR, ** *p* < 0.05 vs. MED CD.

To assess ER stress in patients with CD, rt-PCR was performed in samples after culture for the central genes responsible for UPR activation, EIF2AK3, ERN1, and ATF6. EIF2AK3 encodes the PERK pathway, and it was significantly upregulated in intestinal mucosa from the CD group compared with the control group (*p* = 0.005) (Figure 3A). ERN1, responsible for activating the IRE1 pathway, and ATF6 were not modulated in these patients (Figures 3B and 3C, respectively). This analysis included intestinal samples from 10 CD patients and 6 controls, as mentioned in Table 2 before.

As this is the first study to evaluate the effect of PBA on explant culture from patients with CD, two different concentrations, 5 mM and 10 mM, were assessed to determine the ideal one. PBA 5 mM significantly decreased the expression of EIF2AK3 (*p* < 0.05), whereas PBA 10 mM did not affect the expression of this gene (Figure 3A).

### 3.3. Chaperones and Genes Responsive to UPR Were Blocked with PBA in the Intestinal Mucosa of Crohn’s Disease Patients

**Figure 4:**
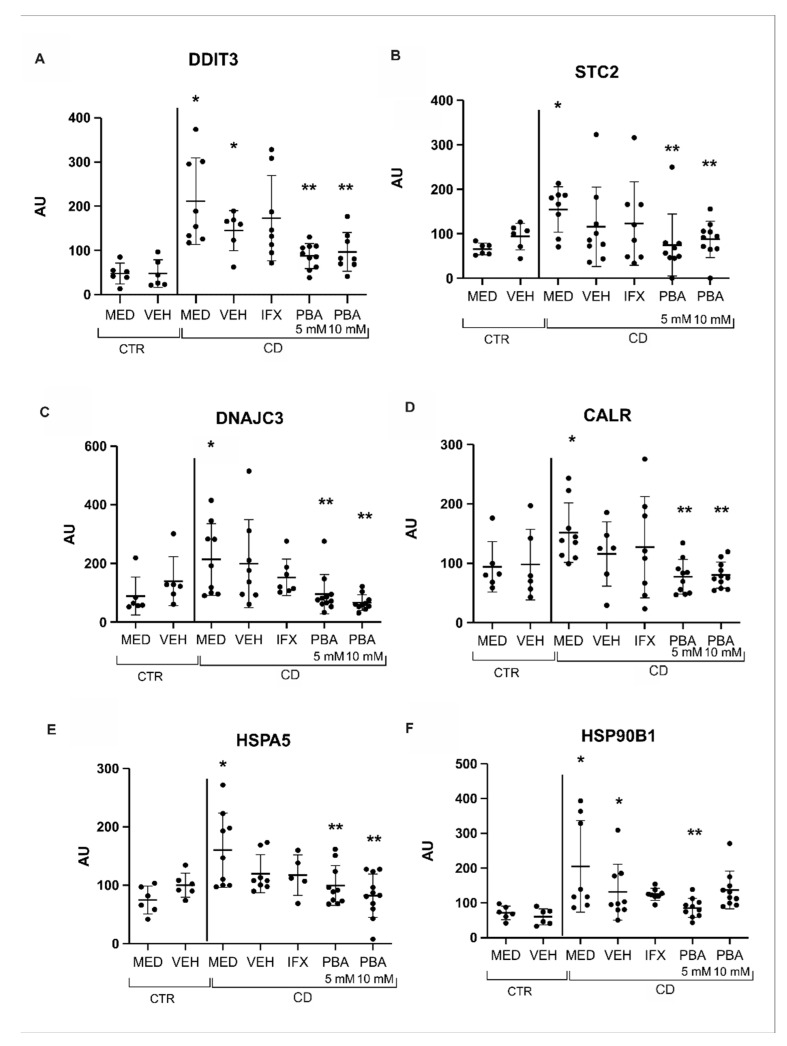
**Activation of chaperones and UPR-related genes and the PBA treatment.** The transcriptional levels of DDIT3 (**A**), STC2 (**B**), DNAJC3 (**C**), CALR (**D**), HSPA5 (**E**), and HSP90B1 (**F**) were significantly increased in the MED CD condition compared to the MED CTR condition. DDIT3, STC2, DNAJC3, CALR, HSPA5, and HSP90B1 transcriptional levels significantly decreased compared to the MED CD condition after PBA treatment. MED = medium, VEH = vehicle, IFX = infliximab, PBA = 4-phenylbutyrate acid, CD = Crohn’s disease, CTR = control. * *p* < 0.005 vs. MED CTR, ** *p* < 0.05 vs. MED CD.

The regulation of chaperones and UPR-related genes is triggered by ER stress activation. Once PBA effectively decreased ER stress, we performed a transcriptional analysis of the central genes related to UPR before and after the treatment with PBA. We observed a significant increase in the expression of DNA damage-inducible transcript 3 (DDIT-3), calcium-regulatory protein stanniocalcin-2 (STC2), Dna J heat shock protein family (DNAJC3), calreticulin (CALR), and HSPA5, which encodes GRP78 and HSP90B1, which encode GRP94. All these genes were significantly decreased after the treatment with PBA (Figure 4A–F).

### 3.4. PBA as an Immune Modulator in Crohn’s Disease

**Figure 5:**
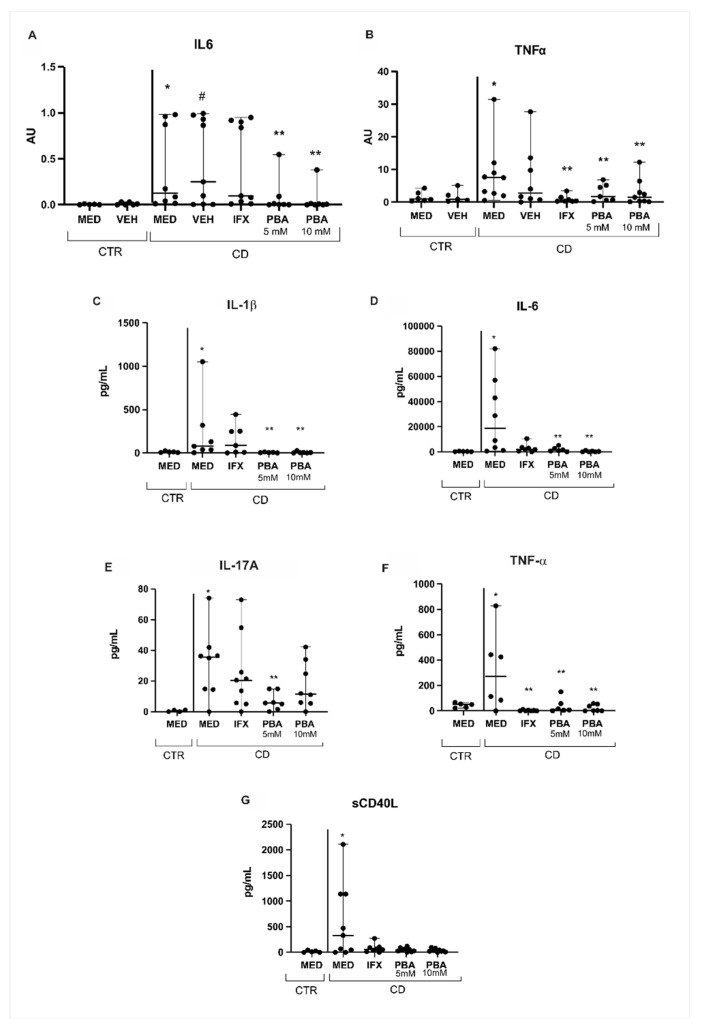
**Transcriptional and protein expression of pro-inflammatory cytokines in Crohn’s disease and the effect of PBA as an immunomodulator**. (**A**) The IL6 transcriptional level was significantly increased in the MED CD condition compared to the MED CTR condition; both concentrations of PBA significantly decreased the IL6 level compared to the MED CD condition. (**B**) The TNFα transcriptional level was significantly increased in the MED CD condition compared to the MED CTR condition; both concentrations of PBA and the treatment with IFX significantly decreased the TNFα level compared to the MED CD condition. (**C**–**G**) There was a significant increase in IL-1β (**C**), IL-6 (**D**), IL-17 (**E**), TNF-α (**F**), and sCD40L (**G**) protein levels in the MED CD condition compared to the MED CTR condition; in addition, a significant decrease in these proinflammatory cytokines in the PBA treatment conditions was observed for almost all cytokines compared to the MED CD condition, except for sCD40L. MED = medium, VEH = vehicle, IFX = infliximab, PBA = 4-phenylbutyrate acid, CD = Crohn’s disease, CTR = control. * *p* < 0.05 vs. MED CTR; ** *p* < 0.05 vs. MED CD, # *p* < 0.05 vs. VEH CTR.

As mentioned, ER stress is prone to be closely involved with an immune imbalance observed in CD. To evaluate whether PBA acts indirectly as an immune modulator, we assessed the transcriptional level of interleukin 6 (IL6) and tumor necrosis factor α (TNFα), two of the central pro-inflammatory cytokines in CD. Both cytokines were upregulated in the MED CD condition compared to the control (Figure 5A,B). There was a significant decrease in both IL6 and TNF-α levels in the CD group for both concentrations of PBA compared to the MED CD condition (Figure 5A,B). In addition, there was a significant difference in the TNF-α level after the Infliximab (IFX) treatment.

In addition, we performed a multiplex assay to quantify the inflammatory profile from the supernatant medium after the explant culture. We observed a significant increase (*p* < 0.05) in the protein expression of IL-1β, IL-6, IL-17, TNF-α, and sCD40L, a soluble member of the TNF-α family (Figure 5C–G) in MED CD condition compared to MED CTR condition. After the treatment of PBA, there was a significant decrease in the expression of all cytokines except for sCD40L in the PBA-treated conditions compared to the MED CD condition (Figure 5C–G). IL-4, IL-10, IL-21, IL-22, IL-23, IL-25, IL-31, IL-33, IFN-γ, and IL-17F were also evaluated, but no statistical differences were observed among the conditions (Supplementary Figure S1).

## 4. Discussion

The management of the pharmacological treatment of CD is a challenge for clinicians in the area [38]. In addition to leading to several side effects, especially with the use of corticosteroids [39], available options, such as biological therapy, represent a considerable rate of non-response [40]. Given this, the in-depth study of other inflammation maintenance pathways, as occurs in ER stress, and the blocking of its activation has become of great value in the scientific literature [41,42].

Although the molecular mechanism that triggers ER stress activation in IBD is not entirely understood, several studies have reported ER stress as one relevant component in the maintenance of immune-mediated disease [18,20,43]. The endoplasmic reticulum (ER) is one of the main intracellular organelles that assists the cell in maintaining its homeostasis. When ER function is impaired, it leads to a state of stress in the ER, and the UPR is activated to restore the physiological state [44].

Several conditions lead to ER impairment, including the overproduction of specific proteins, such as pro-inflammatory cytokines, a feature seen in CD. In patients with active CD, ER cannot process protein overload produced by persistent inflammation in the intestine [45,46]. The presence of unfolded protein in the lumen of ER disattaches BiP protein, also known as GRP78, from PERK, IRE1, and ATF6 receptors, initiating the UPR. ER stress and UPR activation induce apoptosis via DDIT3, protein degradation, and/or the production of chaperones, including STC2, CALR, and DNJAC3, to assist the protein folding process [44]. Younes et al. investigated the therapeutic potential of Tocilizumab, a monoclonal antibody targeting IL-6, in an animal model of ulcerative colitis. Their study comprehensively assessed inflammatory, immunomodulatory, apoptotic, autophagy-related, and ER stress markers, along with clinical parameters such as stool consistency, rectal bleeding, and edema in rats. The findings demonstrated that Tocilizumab downregulated IRE-1 and ATF-6, as well as autophagy-related proteins, including autophagy-related 16-like protein 1 (ATG16L1) and nucleotide-binding oligomerization domain-containing protein 2 (NOD2) [47].

Small molecules with chemical compounds have been reported in the literature to be able to reverse the folding of misfolded proteins [42]. 4-phenylbutyrate (4-PBA) is a low-molecular-weight fatty acid and a non-toxic pharmacological compound with a chaperone-like activity, and it was shown to increase protein folding in liver tissue [48]. Its physicochemical properties enable it to stabilize peptide structures, improving the luminal folding capacity and traffic of aberrant proteins. In an animal model of liver injury, treatment with 4-PBA resulted in the inhibition of ER stress, significantly reducing liver damage. Additionally, 4-PBA lowered levels of DDX3X, a critical factor in immune-mediated liver injury [49]. Thus, PBA may provide a therapeutic approach to block the pathological process induced by protein overload [25]. An animal study showed that oral administration of PBA in DSS-induced colitis mice promoted a decrease in ER stress markers and inflammatory cytokines [18]. Several prior studies [34,35,36,37] have demonstrated that PBA, at a concentration of 5 mM, effectively inhibits markers of ER stress. Building on this evidence, we investigated whether a higher concentration of PBA (10 mM) might yield enhanced inhibitory effects. To our knowledge, no study evaluates the effect of PBA directly on intestinal mucosa from human samples.

The PERK pathway induces the expression of DDIT3, an ER-related apoptosis marker, via the expression of the transcription factor ATF4 [50]. In our study, we observed, via rt-PCR, that CD patients (MED CD condition) presented an upregulation of the EIF2AK3 transcript, responsible for PERK activation, compared to control patients. A study published by Lin and colleagues [51] showed that prolonged PERK activity contributes to the induction of cell death. We also observed an upregulation in the DDIT3 transcriptional level in the CD group compared to the control, which, gathered with the high expression of EIF2AK3, suggests that apoptotic events were occurring in the intestinal mucosa from patients with prolonged activated CD (our patients presented almost 10 years of disease). After the treatment with PBA, we observed a significant decrease in EIF2AK3 and DDIT3 levels compared to the control. This result suggests that PBA is efficacious for blocking ER stress as it modulates the PERK pathway and decreases ER stress-related apoptosis. We verified that PBA 10 Mm did not affect the expression of EIF2AK3, only PBA 5 Mm. The dose–response relationship is complex and depends on several factors. A higher dose does not always produce a more significant effect. The relationship between the dose of a reagent and the effect produced is not always linear. This may occur for several reasons, such as receptor saturation, some physiological responses that have a maximum limit, regardless of the amount of reagent/drug administered, and negative feedback mechanisms that counterbalance the intensity of the response [52,53]. Choi et al. investigated various concentrations of PBA in synovial fibroblasts, up to 80 mM. Concentrations exceeding 20 mM exhibited cytotoxic effects, resulting in decreased cell viability. These findings suggest that higher doses offer less benefit than moderate doses (5–10 mM) [54].

Several preclinical studies have reported that the absence of the IRE1 gene impairs the mucosa barrier, promoting spontaneous colitis [19] or susceptibility to developing colitis in mice [55]. The activation of ATF6 results mainly in an attempt to restore ER homeostasis, leading unfolded protein to degradation [56,57]. Although GRP78 releases PERK, IRE1, and ATF6 once ER stress is initiated, simultaneously, IRE1 and ATF6 responses decrease as UPR persists [58]. As we did not observe a transcriptional regulation for ERN1 and ATF6 genes responsible for activating IRE1 and ATF6 pathways, these results suggest that both pathways were attenuated once the patients in this study presented a prolonged activated disease.

Both ER stress activation and resolution attempts are mediated by glucose-regulated proteins (GRP)-78 and -94, which act as chaperones, helping the ER restore its balance [57]. Therefore, we evaluated whether those UPR mediators were activated, and we observed that the expression of both HSP90B1 and HSPA5 genes, responsible for encoding GRP78 and GRP94, respectively, are transcriptionally upregulated in the CD group (MED CD condition compared to MED CTR condition). In addition, IHC images have also shown positive immunostaining for GRP-94 in cells from the epithelial layer and lamina propria. We also observed a decrease in these chaperones after the treatment with PBA, reinforcing the ER stress blockage with the PBA treatment.

Most studies evaluating ER stress in IBD patients report activating it in epithelial intestinal cells [59,60,61,62,63,64]. IHC images from our study give new insights into the location of ER stress activation. We observed that some UPR markers show positivity, not only in epithelial intestinal cells but also in cells from lamina propria from CD patients. In addition, another study from our group also reported the presence of positive cells for ER stress and UPR markers in both epithelial intestinal cells and cells from lamina propria from intestinal mucosa but not in mesenteric adipose tissue, from patients with CD who underwent a surgical procedure [20].

Moreover, after PBA treatment by the multiplex assay, we showed significant decreases in several pro-inflammatory cytokines, such as IL-1β, IL-6, IL-17, TNF-α, and sCD40L. The diminish of pro-inflammatory cytokines is crucial in improving clinical outcomes and modulating the immune response in CD. Effective treatments can be developed to induce mucosal healing and manage this complex condition by reducing inflammation, restoring immune balance, and targeting specific cytokines [65,66,67].

Concerning the limitations of our study, a formal sample size calculation was not performed, as it was an observational study. Other potential limitations of the study include the small sample size, the absence of in vivo data, and the lack of long-term follow-up to assess the durability of PBA’s effects. Moreover, we did not directly explore whether and how PBA could interfere with protein folding and the degradation of misfolded proteins in our samples. It is more appropriate to study in animal models or in vivo conditions but our results undoubtedly open frontiers for new protocols.

## 5. Conclusions

In conclusion, blocking the activation of the ER stress with the use of PBA was significantly effective in decreasing the expression of the EIF2AK3- and UPR-related genes in the intestinal mucosa of CD patients. In addition, the PBA effect remarkably reduced the protein levels of the central pro-inflammatory cytokines involved in the disease (Figure 5). Therefore, the blockade of ER pathways plays a potential new target in CD therapy, and PBA may constitute a potential drug with another mechanism of action distinct from those already available in clinical practice for CD clinical therapy. Undoubtedly, exploring the long-term effects of PBA treatment, its potential combination with other therapies, and evaluating its effects in animal models of CD are directions for future research in this field.

## Figures and Tables

**Table 1:** Clinical and demographic characteristics of the patients included in the immunohistochemistry analysis.

	CTR	CD
**Number of patients**	7	8
**Gender (male/female)**	3/4	2/6
**Age (median–min/max)**	38.5 years old (28–51)	51 years old (27–57)
**Montreal classification**		
Age of diagnosis (A1/A2/A3)	-----	2/6/0
Location (L1/L2/L3/L4)	-----	2/3/3/0
Behavior (B1/B2/B3)	-----	4/2/2
Perianal disease (yes/no)	-----	3/5
**Disease duration (median–min/max)**	-----	13.5 years (1–21)
**CDEIS (median–min/max)**	-----	6.62 (0–22.1)
**Medications (number of patients)**	-----	Infliximab (1) Adalimumab (3) Azatioprine (5)

CTR: Control. CD: Crohn’s disease. CDEIS: Crohn’s disease Endoscopic Index of Severity.

**Table 2:** Clinical and demographic characteristics of the patients were included in the transcriptional analysis and multiplex assay.

	CTRL	CD
**Number of patients**	6	10
**Gender (male/female)**	2/4	5/5
**Age (median–min/max)**	64 years old (51–69)	36 years old (23–72)
**Montreal classification**		
Age of diagnosis (A1/A2/A3)	-----	2/6/2
Location (L1/L2/L3/L4)	-----	2/1/7/0
Behavior (B1/B2/B3)	-----	5/4/1
Perianal disease (yes/no)	-----	2/8
**Disease duration (median–min/max)**	-----	9.5 years (5–25)
**CDEIS (median–min/max)**	-----	6.18 (1.24–29.5)
**Medications (number of patients)**	-----	Infliximab (4) Vedolizumb (1) Adalimumab (4) Mesalazine (2) Azatioprine (8)

CTR: Control. CD: Crohn’s disease. CDEIS: Crohn’s disease Endoscopic Index of Severity.

## Data Availability

The original contributions presented in this study are included in the article/Supplementary Materials. Further inquiries can be directed to the corresponding author.

## Supplementary Materials

The following supporting information can be downloaded at https://www.mdpi.com/article/10.3390/cells14040270/s1: Figure S1: Protein expression of pro-inflammatory cytokines in Crohn’s disease and the effect of PBA.
