# Supplementary material for: In Vitro Inhibition of Endoplasmic Reticulum Stress: A Promising Therapeutic Strategy for Patients with Crohn’s Disease
Source: Cells. 2025 Feb 13;14(4):270. doi: 10.3390/cells14040270 (PMC11853800; doi:10.3390/cells14040270)
Supplement: Supplementary file 1 [file cells-14-00270-s001.zip › cells-3407081-supplementary.pdf]

cells

MDPI

In *vitro* Inhibition of Endoplasmic Reticulum Stress: A Promising Therapeutic Strategy for Patients with Crohn's Disease

Bruno Lima Rodrigues, Lívia Bitencourt Pascoal, Lívia Moreira Genaro, Leonardo Saint Clair Assad Warrak, Beatriz Alves Guerra Rodrigues, Andressa Coope, Michel Gardere Camargo, Priscilla de Sene Portel Oliveira, Maria de Lourdes Setsuko Ayrizono, Lício Augusto Velloso, and Raquel Franco Leal.

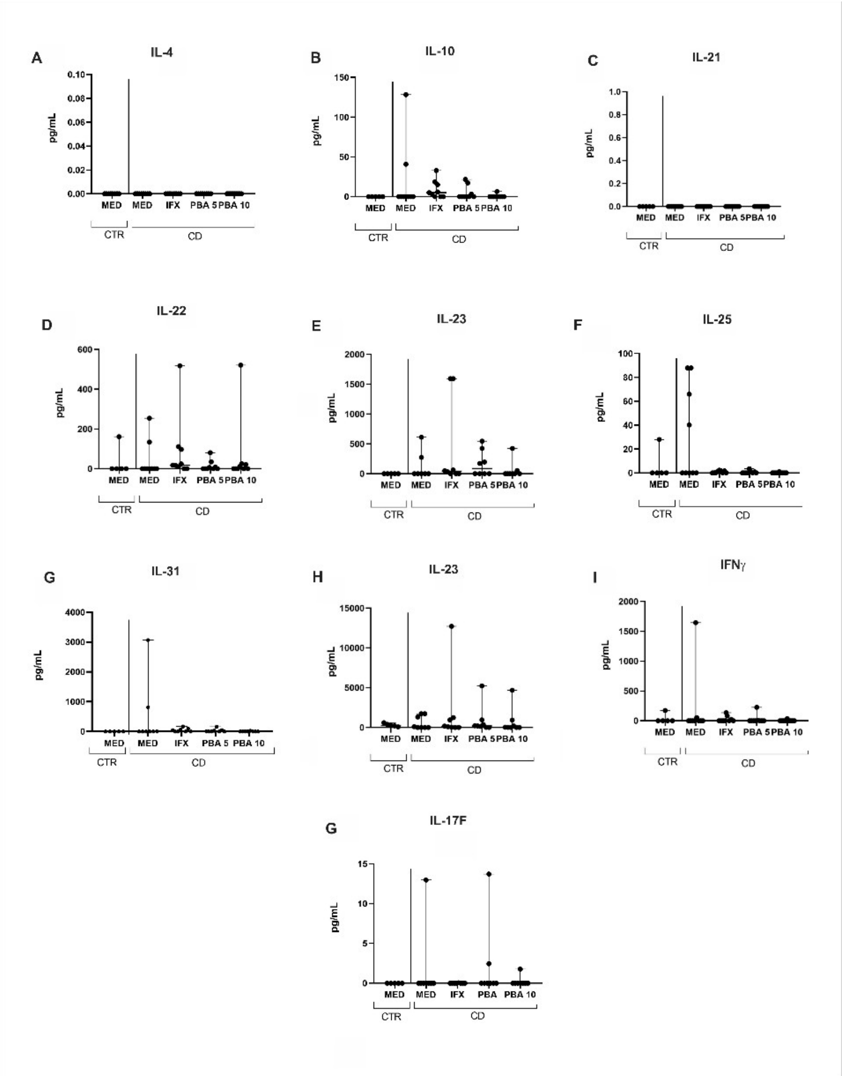

Supplementary Figure S1. Protein expression of pro-inflammatory cytokines in Crohn's disease and the effect of PBA. (A) IL4 , (B) IL-10, (C) IL-21, (D) IL-22, (E) IL-23, (F) IL-25, (G) IL-31, (H) IL-33, (I) IFN-γ, and (J) IL-17F. MED = medium, IFX = infliximab, PBA = 4-phenylbutyrate acid, CD = Crohn's disease, CTR = control. *p<0.05 vs MED CTR; **p<0.05 vs MED CD.
